# Neuroprotective Effects of Myrtenal in an Experimental Model of Dementia Induced in Rats

**DOI:** 10.3390/antiox11020374

**Published:** 2022-02-12

**Authors:** Stela Dragomanova, Stoyan Pavlov, Desislava Marinova, Yordan Hodzev, Maria Cristina Petralia, Paolo Fagone, Ferdinando Nicoletti, Maria Lazarova, Elina Tzvetanova, Albena Alexandrova, Reni Kalfin, Lyubka Tancheva

**Affiliations:** 1 Department of Pharmacology, Toxicology and Pharmacotherapy, Faculty of Pharmacy, Medical University, 9002 Varna, Bulgaria; stela_dragomanova@abv.bg; 2 Department of Biological Effects of Natural and Synthetic Substances, Institute of Neurobiology, Bulgarian Academy of Sciences, Acad. G. Bonchev St., Block 23, 1113 Sofia, Bulgaria; m.lazarova@inb.bas.bg (M.L.); elina_nesta@abv.bg (E.T.); aalexandrova@abv.bg (A.A.); reni_kalfin@abv.bg (R.K.); ltancheva@bio.bas.bg (L.T.); 3 Department of Anatomy and Cell Biology, Faculty of Medicine, Medical University, 9002 Varna, Bulgaria; stoyan.pavlov@mu-varna.bg (S.P.); desi.marinova@mu-varna.bg (D.M.); 4 National Center of Infectious and Parasitic Diseases, 26 Yanko Sakazov Blvd, 1504 Sofia, Bulgaria; y.hodzhev@ncipd.org; 5 Department of Clinical and Experimental Medicine, University of Messina, 98122 Messina, Italy; m.cristinapetralia@gmail.com; 6 Department of Biomedical and Biotechnological Sciences, University of Catania, Via S. Sofia 89, 95123 Catania, Italy; paolo.fagone@unict.it; 7 Faculty of Public Health, Healthcare and Sport, South-West University “Neofit Rilski”, Ivan Mihailov St. 66, 2700 Blagoevgrad, Bulgaria

**Keywords:** myrtenal, experimental dementia, acetylcholine, acetylcholinesterase, antioxidant, neuroprotection, neuromodulation

## Abstract

There is growing attention on natural substances capable of stimulating the cholinergic system and of exerting antioxidant effects, as potential therapeutic agents in Alzheimer’s disease (AD). The aim of the present study is to evaluate the expected neuroprotective mechanisms of myrtenal (M) in an experimental model of dementia in rats. Dementia was induced in male Wistar rats by scopolamine (Sc) administration (0.1 mg/kg for 8 days and 20.0 mg/kg on day 9). The animals were divided into 5 groups (1) Controls; (2) Sc; (3) Sc + Myrtenal (40 mg/kg), (4) Sc + Galantamine (1 mg/kg); (5) Sc + Lipoic acid (30 mg/kg). Changes in recognition memory and habituation were evaluated via the Novel Object Recognition and Open Field tests. Acetylcholinesterase (AChE) activity, ACh levels, and changes in oxidative status of the brain were measured biochemically. The histological changes in two brain regions—cortex and hippocampus, were evaluated qualitatively and quantitatively. Myrtenal improved recognition memory and habituation, exerted antioxidant effects and significantly increased ACh brain levels. Histologically, the neuroprotective capacity of myrtenal was also confirmed. For the first time, we have demonstrated the neuroprotective potential of myrtenal in an experimental model of dementia. Our study provides proof-of-concept for the testing of myrtenal, in association with standard of care treatments, in patients affected by cognitive decline.

## 1. Introduction

Alzheimer’s disease (AD) is one of the most common forms of dementia worldwide. The main features of AD are cholinergic dysfunction and increased oxidative stress, with progressive loss of neuronal structures, associated with accumulation of abnormal proteins [1], mitochondrial dysfunction [2], and excitotoxicity [3].

AD is characterized by cholinergic neurons loss in the forebrain, responsible for attention and memory status, and in the cortex and hippocampus [4,5,6].

In AD, the activity of choline acetyltransferase (ChAT), the enzyme responsible for acetylcholine synthesis, is decreased, while acetylcholinesterase (AChE) activity, which breaks down Ach, is increased. Hence, the current approach to improve memory impairment is the inhibition of acetylcholinesterase activity, by using drugs, such as Galantamine, Rivastigmine, and Donepezil.

Oxidative stress is also associated with major pathological processes underlying AD and correlates with the severity of the neurodegenerative changes [7,8]. It is believed that preventing reactive oxygen species (ROS) formation could slow both the onset and progression of AD. Therefore, antioxidant agents may represent a successful approach in the prevention and treatment of AD [9]. Indeed, some terpenoids, such as ginsenosides, ginkgolides, cannabinoids, and oleanolic and ursolic acid, exert potential effects on neurodegenerative disorders, including AD, affecting the activity of acetylcholinesterase, reducing Aβ-aggregation and oxidative stress, etc. [10].

Myrtenal is a monoterpenoid commonly found in plants, including *Myrtus communis*, *Artemisia* spp., *Origanum majorana*, *Origanum vulgare*, *Glycyrrhiza glabra*, *Rosmarinus officinalis*, *Thymus* spp., *Salvia officinalis*, *Lavandula* spp., as well as in bee glue (propolis). In plants, myrtenal is metabolically linked to α-pinene [11], which has been shown to ameliorate memory and learning deficits in an animal model of amnesia, by protecting the acetylcholine signaling and by promoting the antioxidant defense of the brain [12]. Numerous beneficial effects of Myrtenal have been already described, e.g., bronchodilatory, anti-inflammatory, antiplatelet and anti-hemolytic, as well as antibacterial (against G (+) pathogens) [13,14,15,16].

The effects of myrtenal administration in control rodents (mice and rats) have been previously explored [17]. In vitro, myrtenal has been shown to exert an inhibitory effect on AChE activity [18], suggesting its beneficial effect on memory impairment. Our previous studies have also demonstrated that myrtenal exerts a protective effect in an experimental model of Parkinson’s disease induced in rats [19].

However, there are no data in the literature, about the effects of myrtenal in experimental animal models of dementia. The aim of the present study was to evaluate the potential neuroprotective mechanism of myrtenal in an experimental model of dementia, induced by Scopolamine in rats.

## 2. Materials and Methods

### 2.1. Chemicals

Alfa-lipoic acid (LA) (Thoictic acid—solution for injection 600 mg/50 mL) Thiogamma Turbo-Set (Wörwag pharma GmbH & Co.KG, Galwer Strasse 7, 71034 Böblingen, Germany), Lot: 16J171; Galantamine (Gal) (solution for injection 10 mg/mL, 1 mL ampules Nivalin) Sopharma, Lot: 11215; (−)-Myrtenal 98%—ACRÔS Organics, Lot: A0363097; Scopolamine (Sc)—ACRÔS Organics, Lot: A0354964; Sodium Chloride 0.9% solution, BRAUN.

### 2.2. Docking Study

A docking analysis was performed in order to evaluate myrtenal’s affinity to acetylcholine esterase (AChE). Gal was used as a reference. The crystallographic structure of the complex between acetylcholinesterase and Gal with PDB code 4ey6 was used. Docking was performed on a flexible ligand and rigid protein. The binding site was set within a radius of 15 Å from the crystallographic structure of Gal. The structural water, HOH846, in the active center is preserved. One hundred poses for myrtenal and Gal were created using the stochastic method for studying the conformational space—a genetic algorithm implemented in the GOLD program. To evaluate the obtained complexes, the ChemPLP evaluation function was used. The analysis was performed using the GOLD v.5.2.2 program.

### 2.3. Experimental Animals

The experiments were carried out on male adult Wistar rats (180 ÷ 220 g). The animals were kept under standard laboratory conditions in plastic cells—12 h light/dark cycle, unrestricted access to drinking water and food for rodents, provided optimum temperature, humidity, and indoor ventilation. The experimental procedures were carried out in accordance with the rules for working with experimental animals of the Committee on Ethics of Bulgarian Food Safety Agency and with National laws and rules (Ordinance No. 20 of 1 November 2012 on the minimum requirements for the protection and welfare of experimental animals and requirements to establishments for their use, rearing and/or delivery, effective from 1 January 2013, issued by the Ministry of Agriculture and Food, Prom. SG issue 87 of 9 November 2012) based on the European Directive and in accordance with the rules for working with laboratory animals of the Ethics Committee of the Institute of Neurobiology at the Bulgarian Academy of Sciences.

### 2.4. Experimental Model of Dementia

In an attempt to recreate the nonlinear progression of neurodegenerative impairment, our team has previously developed a new Sc-induced model of dementia in rats. This new method was previously implemented and validated via behavioral, biochemical, and histological assessments [17]. Briefly, this model consists of the administration of low-dose Sc (0.1 mg/kg) for 8 days, followed by single high-dose Sc (20 mg/kg), on the 9th day.

### 2.5. Drug Treatment and Experimental Design

The animals were divided into 5 groups (*n* = 18 each): (1) Controls; (2) Sc; (3) Sc + Myrtenal, (4) Sc + Gal; (5) Sc + LA. Control group received intraperitoneal (i.p.) saline injection (0.9% NaCl, 0.5 mL/100 g bw). Myrtenal (40 mg/kg) and two reference compounds—the antioxidant LA (30 mg/kg) and cholinergic enhancer Gal (1 mg/kg) were applied i.p simultaneously with Sc for 9 consecutive days. Sc was injected as a scopolamine hydrobromide dissolved in distilled water, myrtenal as an emulsion in an effective dose of 40 mg/kg according to our previous data [17].

Animals from all groups were submitted to two behavioral tests (Novel object recognition and Open field), carried out from 9 a.m. to 12 a.m. One hour after the last behavioral test the animals were euthanized via mild CO_2_ inhalation. Two main brain structures related to memory (prefrontal cortex and hippocampus) were separated for histological analysis and brain AChE activity evaluation. The antioxidant and neuromodulatory capacity of myrtenal were carried out in whole brain homogenates according to the experimental protocol.

### 2.6. Behavioral Tests

The novel object recognition test was used for the evaluation of recognition memory [20,21]. Using the Open field experimental setup, the time that animals spent examining the unknown (new) objects over a period of 5 min was tracked. The experimental platform is a white plastic box (100 × 100 × 40 cm) with a floor divided into equal squares (20 × 20 cm). The experimental protocol includes three steps: day 1: habituation phase—for a period of 5 min the animals are allowed to freely study the territory without the presence of the objects; day 2: recognition phase—one object was presented to the rats for a period of 5 min; day 3: test phase—two objects were presented to the rats for a period of 5 min. One from the previous day (known) and a new one (unknown).

Each rat test trial was video recorded and the time spent in exploration of each object was calculated. Discrimination index (DI) calculation was used for the evaluation of recognition memory changes among the groups. DI = t (new)/t (old + new), where (t) is a time spend for explore new or old object. A DI > 0.5 means that the subjects remember and distinguish objects, while a DI < 0.5 indicates an impaired recognition memory.

The test was performed three times: before all treatments, on day 8 after multiple low dose scopolamine administration, and on day 9 after single high dose scopolamine administration.

The open field test was used for the evaluation of general activity [22] and for behavior exploration, indicative of habituation impaired [23]. It was conducted in a white plastic box (100 × 100 × 40 cm) with the floor divided into equal squares (20 × 20 cm). Each animal was placed in the center of the board and the number of crossing lines (with four paws) were recorded for 5 min.

The test was performed twice: before treatment (initial training, IT) and on day 10 (24 h after the last scopolamine treatment). Results were presented as a delta cross index—differences between the number of line crossings on day 10 versus the initial training level (IT).

### 2.7. Biochemical Studies

#### 2.7.1. Evaluation of Oxidative Brain Status

Post-nuclear brain homogenate was used to measure the following biochemical parameters: total glutathione (GSH), measured according to Tietze [24] and expressed in ng/mg protein, with glutathione oxidized (GSSG) as a reference standard; lipid peroxidation products, determined by the amount of thiobarbituric acid reactive substances (TBARs) formed in fresh biological preparations [25]. The post-nuclear homogenates of the brain structures (mg protein/mL) in 0.15 M KCl-10 mM potassium phosphate buffer, pH 7.4, were heated for 15 min at 100 °C in the presence of 2.8% trichloracetic acid + 5N HCl + 2% thiobarbituric acid in 50 mM NaOH (2:1:1 *v*/*v*) for color developing. The absorbance was read at 532 nm against an appropriate blank. The values were expressed in nmol Malone dialdehyde (MDA) per mg protein, with a molar extinction coefficient of 1.56 × 10^5^ M^−1^cm^−1^. Catalase (CAT) activity was determined according to Aebi [26]; the enzyme activity was expressed as ∆ E_240_/min/mg protein. Cu, Zn-superoxide dismutase (SOD) activity, determined according to Beauchamp and Fridovich [27], was expressed in U/mg protein (one unit of SOD activity is the amount of the enzyme producing a 50% inhibition of Nitroblue tetrazolium reduction). Glutathione peroxidase (GPx) activity was measured by the method of Gunzler et al. [28] and was expressed in nmol NADPH oxidized per minute per mg protein, with a molar extinction coefficient of 6.22 × 10^6^ M^−1^cm^−1^. Protein content was measured by the method of Lowry et al. [29].

#### 2.7.2. Evaluation of Brain AChE Activity

The Ellman protocol (1961) was used for the determination of acetylcholinesterase activity in the two brain structures, cortex, and hippocampus [30]. Samples (supernatants) were added to a solution containing 1.0 mM acetylthiocholine (AcSCh), 0.1 mM 5,5’-dithio-bis (2-nitrobenzoic acid) (DTNB) and 100 mM phosphate buffer (pH 8.0). Incubate for 5 min at 37 °C. The formation of a yellow color in the reaction of thiocholine with dithiobisnitrobenzoate ions (DTNB) was detected spectrophotometrically (λ = 412 nm). The results are expressed as AChE µmol min/g protein.

#### 2.7.3. Evaluation of Brain ACh Content

A method described by Fellman (1969) [31] and modified by Kozlov (1999) [32] was used to determine the concentration of acetylcholine in the brain. After euthanasia of the experimental animals, the brains were separated and placed on ice, followed by washing and homogenization in phosphate buffer (pH 7.4). The homogenates were centrifuged for 15 min at 5000 rpm. As a result of the interaction of acetylcholine from the biological sample with hydrazine and salicylic aldehyde, a fluorophore (specific fluorescence in dark red) was formed, allowing its determination. After the incubation time, the specific fluorescence of the resulting compound was determined at λ excit. = 410 nm and λ fluor. = 540 nm. There was a relationship between the fluorescence intensity and the concentration of the test substance (ng/g tissue), in which case the standard straight-line method was used.

### 2.8. Histological Analysis

Histological analyses were performed in the two brain structures related to learning and memory, namely the cortex and hippocampus. The brains from three (per group) randomly selected animals were fixed in 4.5% paraformaldehyde in phosphate-buffered saline (pH 7.4) for 24 h and embedded in paraffin for routine sectioning. Serial sections of the brain hemispheres (10 µm thickness) were stained with Cresyl-violet after Kluwer-Barrera. The stained sections were captured using a Zeiss AxioImager Z2 microscope imaging system (Carl Zeiss Microscopy GmbH, Oberkochen, Germany) outfitted with a Zeiss Neo-Fluar objective (v20x; NA = 0.5) and AxioCam MR rev.3 camera at a lateral resolution of the obtained images of 0.3225 µm/px. Morphological assessment of the signs of degeneration (qualitative analysis) was performed by two independent investigators in multiple brain sections of each animal.

The image normalization and processing were performed in ImageJ/FIJI software (v.1.43). The images were converted to grey scale and the grey values were transformed to noncalibrated image density according to Equation (1):

(1) $$OD=-\log_{10}(\frac{GV_{px}}{GV_{max}}),$$

where *OD* is the calculated optical density, $GV_{px}$ is the grey value of the corresponding pixel (a measure for the transmitted light at that location) and $GV_{max}$ is the maximal grey value of the background (an estimate of the incident light). In the transformed images, the *OD* is proportional to the light absorbed by the dye. Only pixels with an *OD* of at least 0.097 were included in the subsequent analysis, thus effectively considering only regions of the section that absorb at least 20% of the incident light. This threshold was applied to quantify the stained area and to produce countable masks of the stained profiles. The quantitative analysis of Cresyl-violet stained preparations included: the determination of the average number of stained profiles per unit area (density). Additionally, the mean density (number/mm^2^) of the stained profiles was further analyzed conditional on the particle size: the stained profiles were divided into two classes: “small” profiles with a caliper diameter ≤6.5 µm and “large” profiles with diameter >6.5 µm. The rationale behind this approach was that the neurons have predominantly larger perikarya with an abundance of Nissl bodies and thus will produce stained profiles with a larger area. We have chosen a relatively low caliper of 6.5 µm to account for the probability of the section passing through different portions of the cellular somata. As the glial cells were presented by their stained nuclei (usually there are no Nissl bodies in glial cells), most of the profiles belonging to glia will have sizes smaller than this caliper. On the contrary, profiles with neuronal origin will be expected to be larger than the average nuclear size.

### 2.9. Statistical Analysis

The results are expressed as the means ± the standard error of the mean (SEM). Statistical analyses of the data were performed by one-way analysis of variance (ANOVA). Differences were considered significant at *p* < 0.05.

Quantitative analysis of histopathological studies was performed using R language and environment for statistical computing (version 3.5.2, July 2018, used libraries: tidyverse, car, MASS, lme4, lmerTest, pbkrtest, emmeans simr [33,34,35,36,37,38,39,40,41]). The mean number of profiles per unit area (mm^−2^) was compared between groups of animals using a mixed effects generalized linear model fitted to a negative binomial distribution, which is more appropriate for counting data than the assumption of normality. This analysis is equivalent to one-way ANOVA but takes into account the subjects as a source of random variation and its assumptions fit the data better. Post hoc comparisons between the model estimates were performed with Holm adjustment, where applicable. Results are expressed as model estimated means within their 95% confidence intervals.

### 2.10. Principal Component Analysis

Evaluation of the neuroprotective properties of myrtenal on the proposed Sc-induced model in rats was performed by exploratory principal component analysis (PCA) on standardized (z-transformed) data from animals divided into five groups—Controls, Sc, Sc + Myrtenal, Sc + Gal, and Sc + LA. Missing values were substituted with group mean prior to the standardization. All calculations and graphs produced for the PCA study were performed on the IBM SPSS 19 software. Additionally, hierarchical cluster analysis (centroid clustering method, squared Euclidean distance) was performed on extracted PCA component scores for the first two principal components in order to confirm clustering.

## 3. Results

### 3.1. Effects of Myrtenal on Demented Rats Behavior

#### 3.1.1. Recognition Memory Status

**Figure 1:**
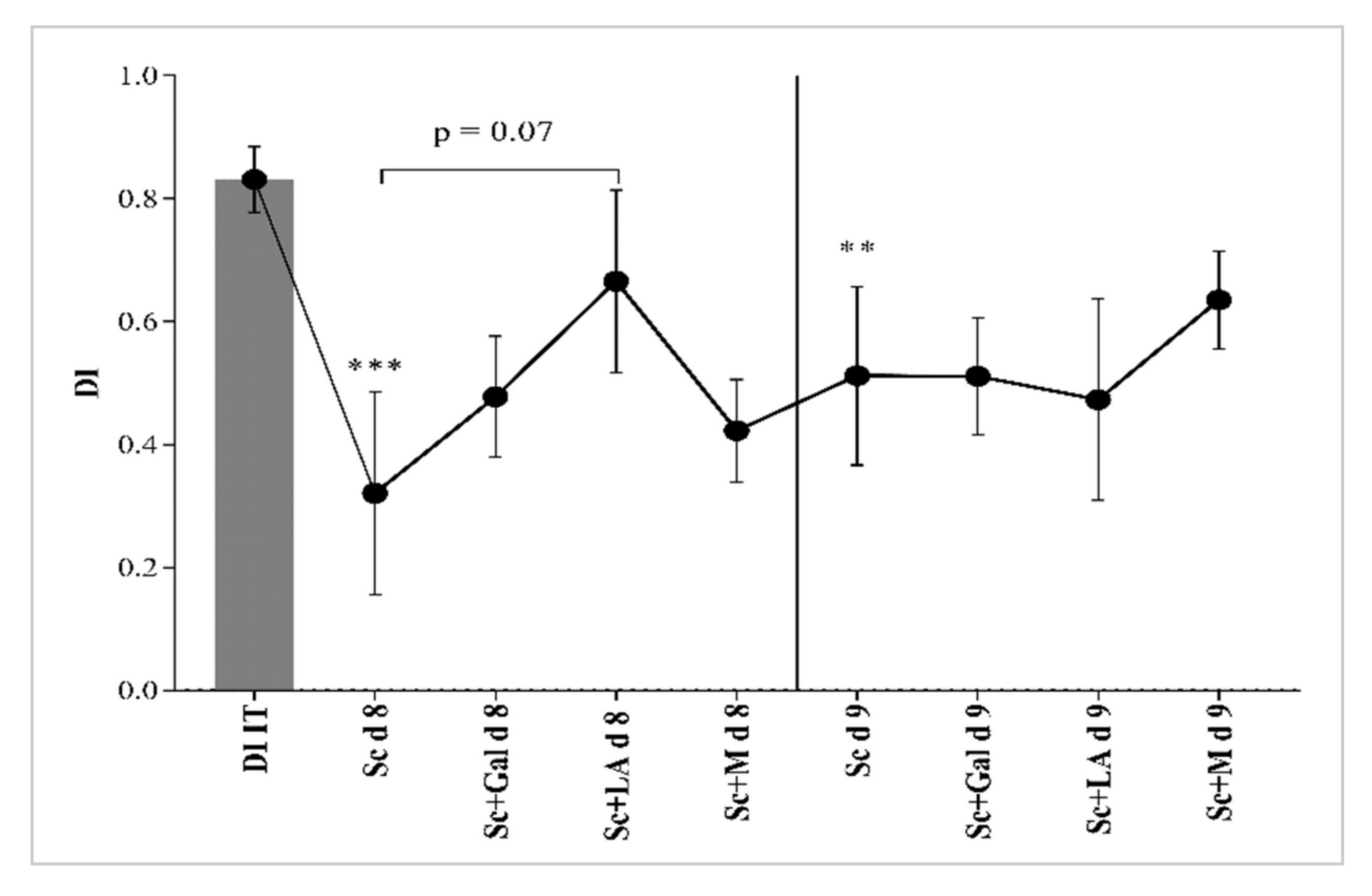
Effects of myrtenal (M) (40 mg/kg) on recognition memory in rats with scopolamine-induced dementia; LA (30 mg/kg) and Gal (1 mg/kg, i.p.) were used as referents. Data are expressed as the mean ± SEM (*n* = 18). *** *p* < 0.001, ** *p* < 0.01 vs. IT.

NOR was made three times: before all treatments (IT), on the 8th day, after multiple low dose Sc administration, and on the 9th day, after single high dose Sc administration. The effects of myrtenal and two reference compounds, Gal and LA, on the recognition memory state were assessed by calculating the discrimination index (DI) (Figure 1).

The mean value of DI of all animals tested before treatment (IT value) was 0.83. Eight days after low-dose Sc (0.1 mg/kg) administration, DI in the Sc group was 0.32 (*p* < 0.001). This reduction is indicative of memory impairment. In the two reference compound groups, Sc + Gal and Sc + LA, DI was 0.48 and 0.67, respectively, which corresponds to a 50% and 107.5% increase, as compared to Sc-treated animals.

A trend for memory improvement was observed also in the Sc + Myrtenal group, where the value of the DI was 0.42, i.e., 31.25% higher than the Sc group.

After the administration of high-dose Sc (20 mg/kg) on the 9th day, the DI value in the Sc-treated animals was 0.5 (*p* < 0.01 vs. Controls). In the Sc + Gal and Sc + LA groups, the DI value was 0.5, indicating a lack of effect on memory improvement. On the other hand, in the Sc + Myrtenal-treated animals, a DI value of 0.635 was observed, which corresponds to a 27% increase as compared to the Sc group.

#### 3.1.2. Effects of Myrtenal on Exploratory Activity

**Figure 2:**
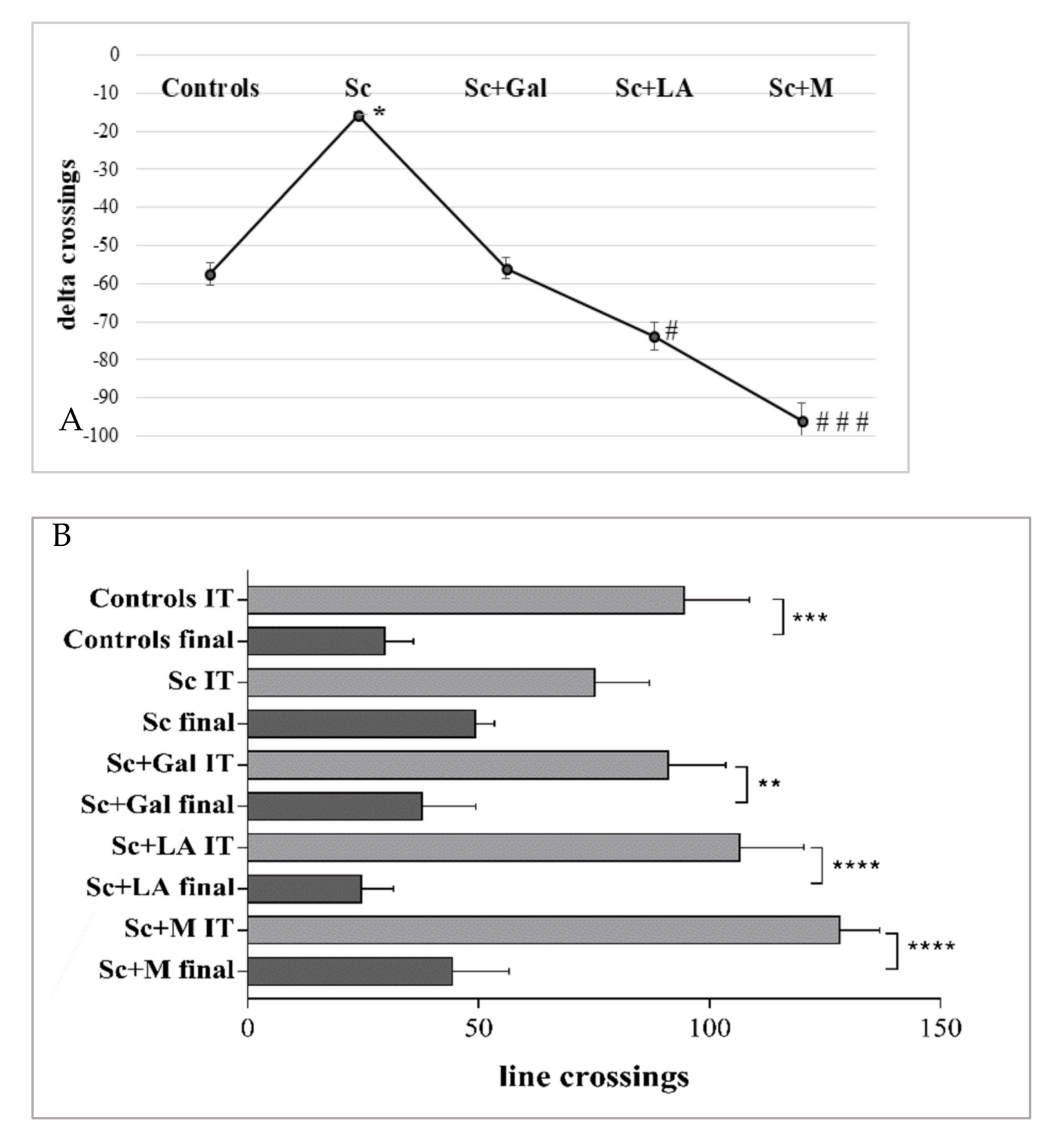
Effect of Myrtenal (M) (40 mg/kg) on exploratory activity in rats with scopolamine-induced dementia. LA (30 mg/kg) and Gal (1 mg/kg) were used as referents. Data are expressed as the mean ± SEM (*n* = 18); (**A**) Delta crossings—difference in line crossings on day 10 vs. initial training (IT); * *p* < 0.05 vs. Controls, ^#^ *p* < 0.05, ^###^ *p* < 0.001 vs. Sc group. (**B**) Line crossings number: ** *p* < 0.01, *** *p* < 0.001; **** *p* < 0.0001 vs. initial training (IT) in the respective group.

The effect of myrtenal on exploratory activity, as a reflection of the changed habituation pattern of dementia rats, was evaluated via the open field test. The test was made two times—before all treatment (initial training, IT) and on day 10 (24 h after the last scopolamine treatment). Results were presented as a delta cross index—differences between the number of line crossings on day 10 versus the initial training level (IT) (Figure 2A) and as a line crossings number (Figure 2B).

In the open field test, control animals showed a statistically significant decrease in line crossing numbers, on day 10 as compared to IT (*p* < 0.001) (Figure 2B). The reduction was by 59.5% (*p* < 0.05) and this is normal expected behavior. Sc-treated animals showed anxiety, confusion, and not significantly changed general activity as compared to the IT of the same group (Figure 2B), which is an indication of an impaired habituation pattern. The delta cross index in this group was increased 3 times in comparison to the control (*p* < 0.05) (Figure 2A).

In the Sc + Gal and Sc + LA groups, the line crossing numbers on day 10 were decreased by 56.7% (*p* < 0.01) and 69.5% (*p* < 0.0001), respectively, as compared to the IT (Figure 2B). The delta crossing index in these groups was also decreased as compared to the Sc-treated animals (Figure 2A).

In the Sc + Myrtenal group, we observed the highest reduction (by 65.5%) of line crossing numbers on day 10, (*p* < 0.0001) as compared to IT (Figure 2B), which means the best-preserved habituation pattern capacity. The decrease in delta cross index was also the biggest—more than 6 times lower (*p* < 0.001) compared to the Sc group (Figure 2A).

### 3.2. Effects of Myrtenal on Brain Oxidative Status in Dement Rodents

**Figure 3:**
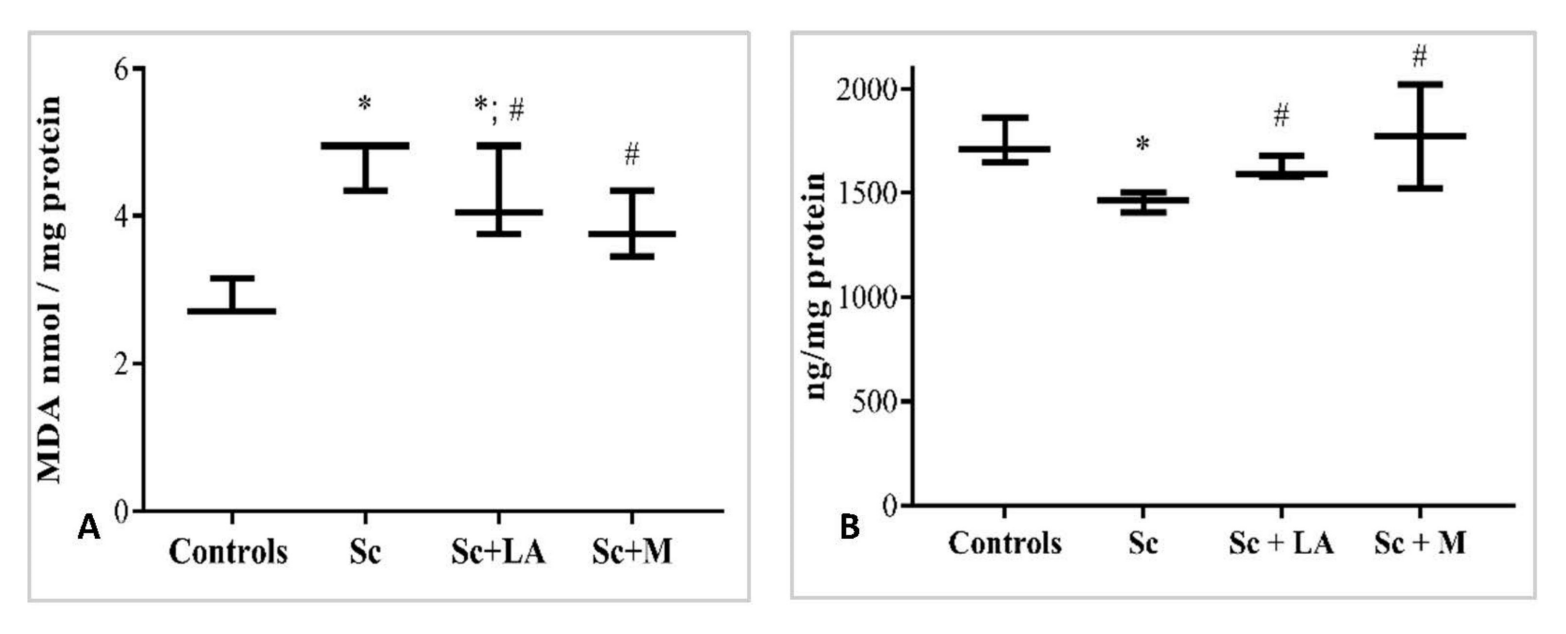
Effects of myrtenal (M) (40 mg/kg) on brain oxidative status in rats with scopolamine-induced dementia (dose modification)—determination of LPO product content (**A**) and GSH levels (**B**); LA (30 mg/kg) was used as a reference. Data are expressed as the mean ± SEM (*n* = 5); * *p* < 0.05 vs. Controls; ^#^ *p* < 0.05 vs. Sc. group.

**Figure 4:**
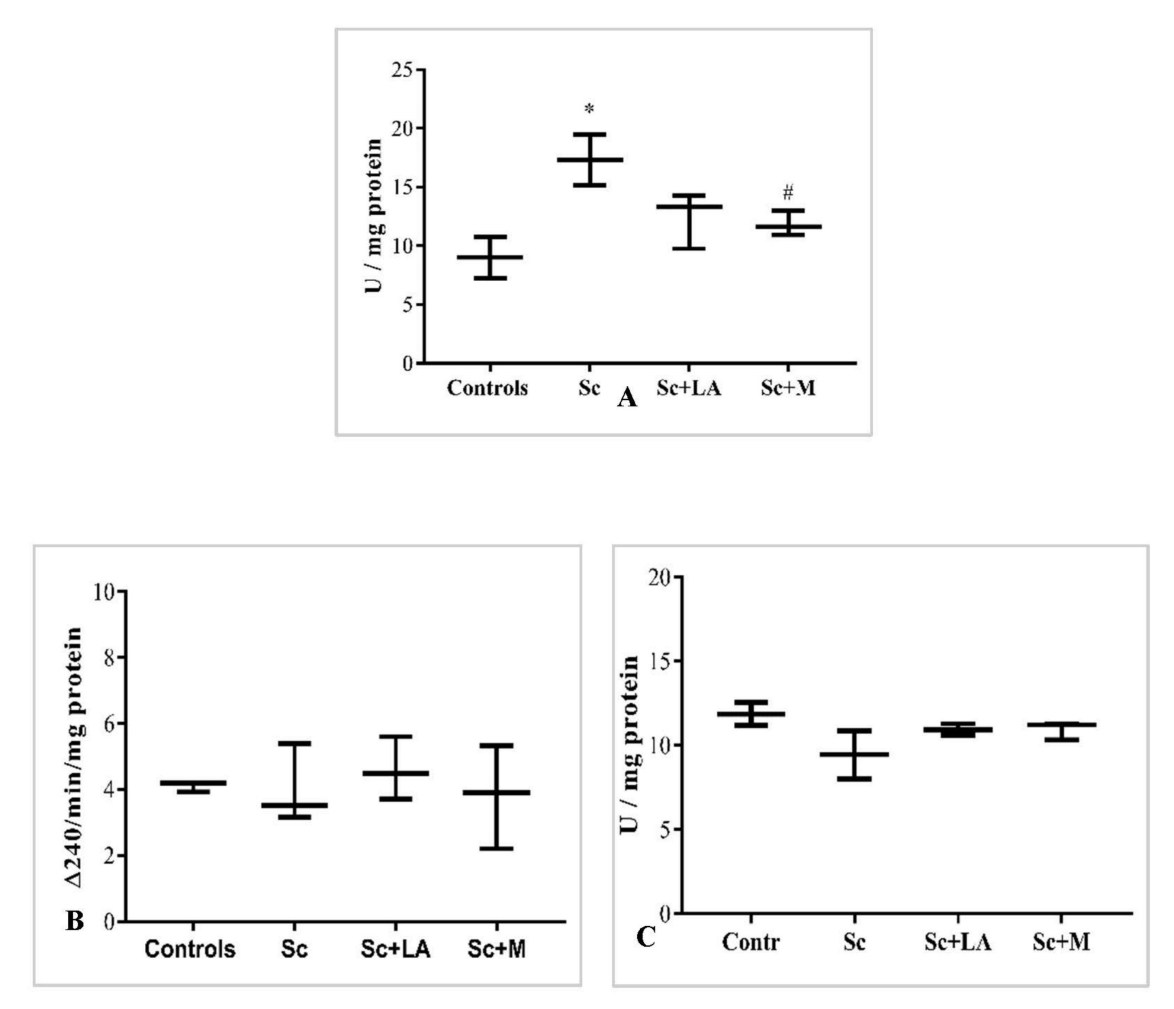
Effect of myrtenal (M) (40 mg/kg, i.p.) on the brain SOD (**A**), CAT (**B**), and GPx (**C**) activity in rats with scopolamine-induced dementia; LA (30 mg/kg) was used as a referent. Data are expressed as the mean ± SEM (*n* = 5); * *p* < 0.05 vs. Controls, ^#^ *p* < 0.05 vs. Sc. group.

The antioxidant properties of myrtenal in the experimental model of dementia were evaluated by determining the levels of LPO products, tGSH content (Figure 3), and activity of some main antioxidant enzymes in the brain—SOD, CAT, and GPx (Figure 4).

Our results showed that multiple Sc treatments enhanced the levels of some oxidative stress parameters in the brains of dementia rats. In comparison to the control, the amount of LPO products was increased by 66.67% (*p* < 0.05) (Figure 3A), tGSH content was decreased by 16.3% (*p* < 0.05) (Figure 3B), and SOD enzyme activity was increased by 45.5% (*p* < 0.05) (Figure 4A). The activities of CAT and GPx (Figure 4B,C) were not significantly changed by Sc.

In Myrtenal-treated animals, the Sc-induced brain oxidative stress was significantly reduced: LPO products were decreased by 19% (*p* < 0.05) (Figure 3A), tGSH content was increased by 14.8% (*p* < 0.05) (Figure 3B), and SOD activity was restored to the control level (*p* < 0.05) (Figure 4A). The antioxidant effect of myrtenal was comparable with that of the reference compound, LA.

### 3.3. Effects of Myrtenal on Brain Cholinergic Transmission in Demented Rodents

#### 3.3.1. Docking Studies

**Figure 5:**
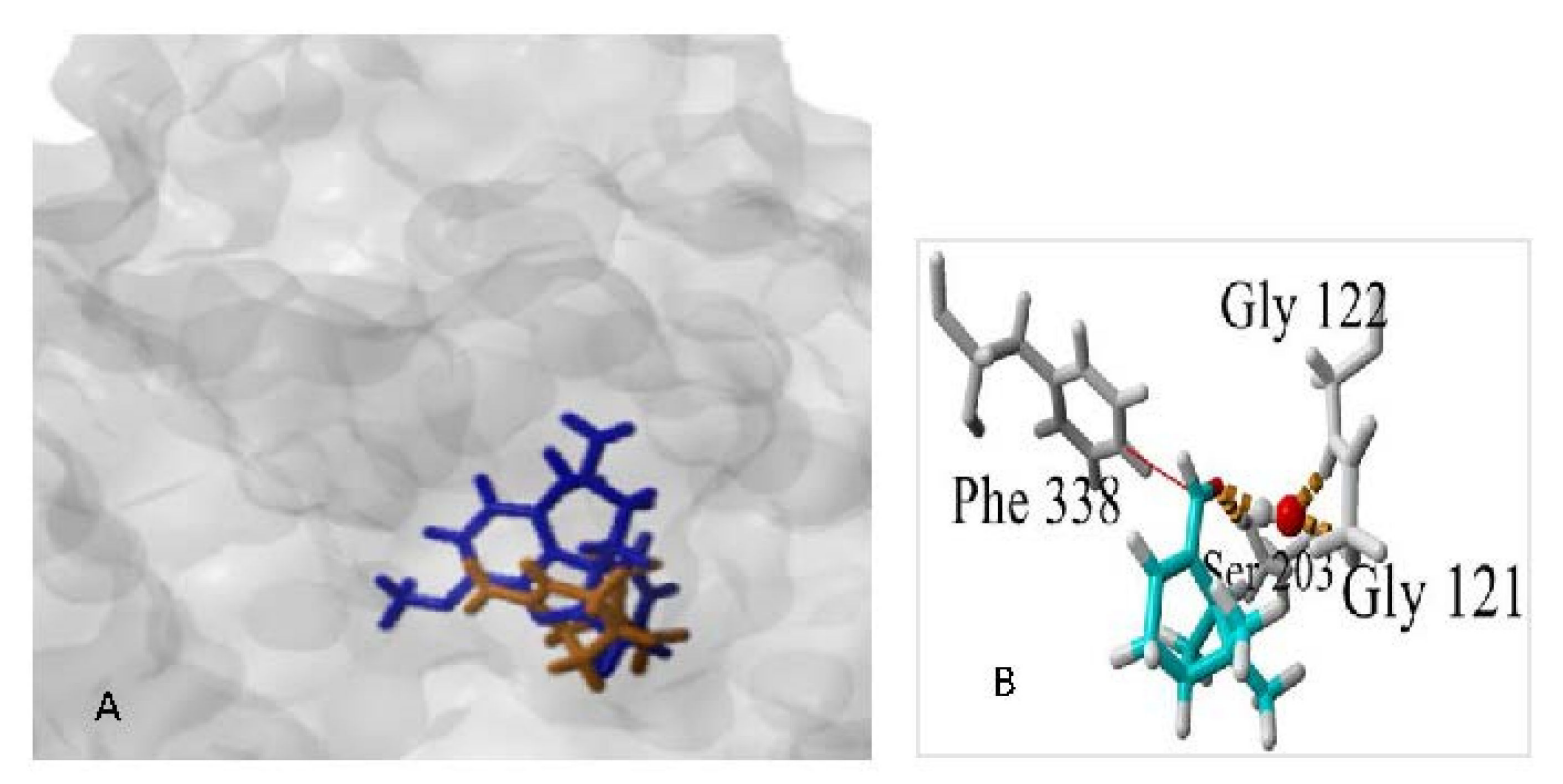
Superposed galantamine (dark blue) and myrtenal (orange) in the active center of AChE (**A**); myrtenal intermolecular interactions in the active center; (**B**) hydrogen bonds are represented by orange disconnected lines; p–π interaction—by a red line, structural water molecule in the active center—by spheres and rods.

Myrtenal’s ability to affect AChE activity was evaluated in comparison to Gal, which was used as a reference for anticholinesterase activity inhibition (Figure 5).

The docking results showed that myrtenal forms a hydrogen bond to Ser203 from the catalytic center at the binding point, as well as a hydrogen bond to the structural water, which serves as a bridge between the enzyme and the ligand, through a network of hydrogen bonds. The Csp2 carbon atom in the aldehyde group of the test substance is involved in p–π interactions with Phe338 amino acid residue from the anion pocket at the binding site. We concluded that the interaction of myrtenal with the catalytic pocket of AChE is weaker than that of Gal.

#### 3.3.2. In Vivo Effect of Myrtenal on Brain AChE Activity

**Figure 6:**
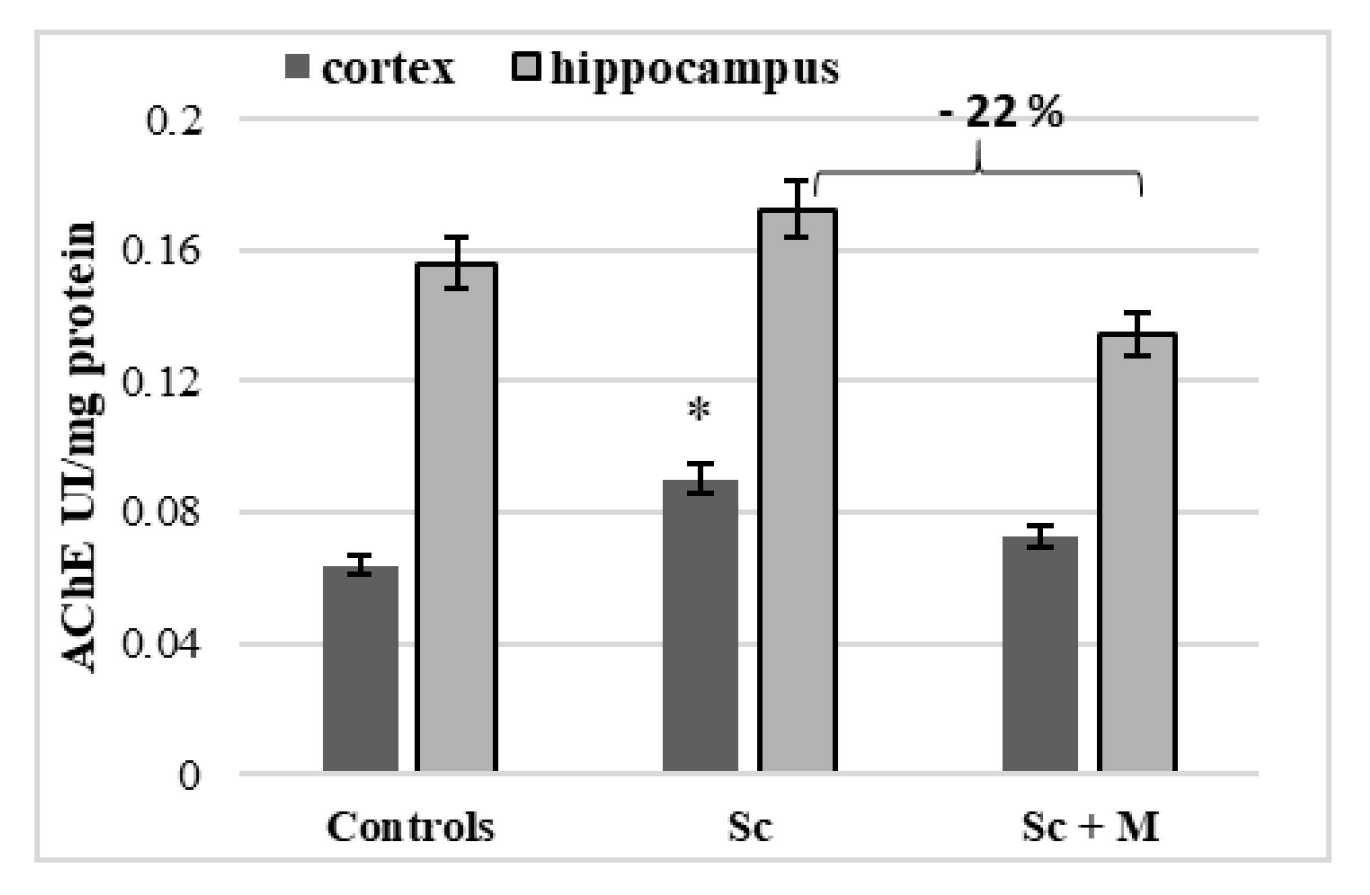
Effects of myrtenal (40 mg/kg) on brain cortex and hippocampus AChE activity in rats with scopolamine-induced dementia. Data are expressed as the mean ± SEM (*n* = 5); * *p* < 0.05 vs. Controls.

The effect of myrtenal on acetylcholinesterase activity was evaluated in brain homogenates from the cortex and hippocampus of rats (Figure 6).

Sc administration increased AChE activity both in the cortex (by 41%, *p* < 0.05) and in the hippocampus (by 10.6%, *p* = n.s.) vs. control levels. In myrtenal-treated animals, the activity of AChE was not significantly decreased by 19.4% in the cortex and by 22% in the hippocampus, in comparison to the Sc-treated animals.

#### 3.3.3. Effect of Myrtenal on Brain ACh Levels

**Figure 7:**
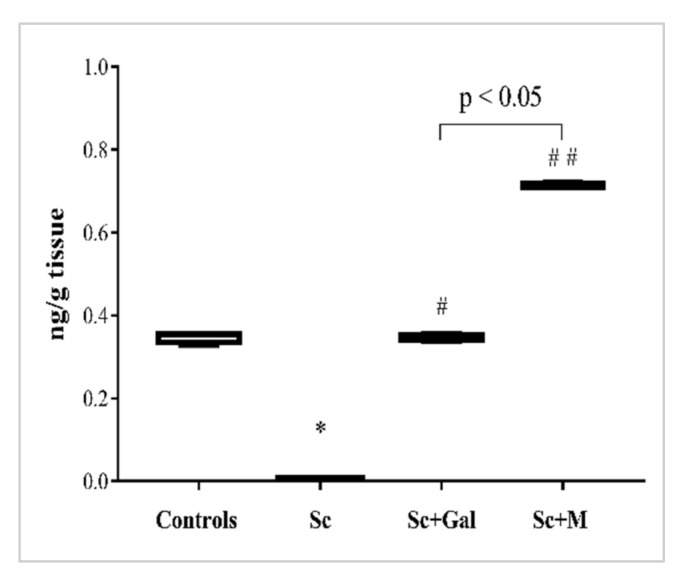
Effect of myrtenal (M) (40 mg/kg) on brain ACh levels in rats with scopolamine (Sc)-induced dementia; Gal (1 mg/kg, i.p.) was used as a referent. Data are expressed as the mean ± SEM (*n* = 5). * *p* < 0.05 vs. Controls; ^#^ *p* < 0.05, ^##^ *p* < 0.01 vs. Sc group.

Our results showed that multiple Sc administrations significantly reduced ACh brain levels. The reduction was more than ~25-fold (*p* < 0.05) as compared to the control (Figure 7).

In Sc + Gal-treated animals, the brain ACh levels were restored to the levels of the control animals. Gal was used as an anti-cholinesterase reference compound.

In the Sc + Myrtenal group, the brain ACh content was significantly increased: 104.9% (*p* < 0.05) vs control, 105.3% (*p* < 0.05) vs Gal and more than 50-fold (*p* < 0.01) as compared to the Sc-treated animals.

#### 3.3.4. ACh Content/AChE Activity Ratio in the Brain

**Figure 8:**
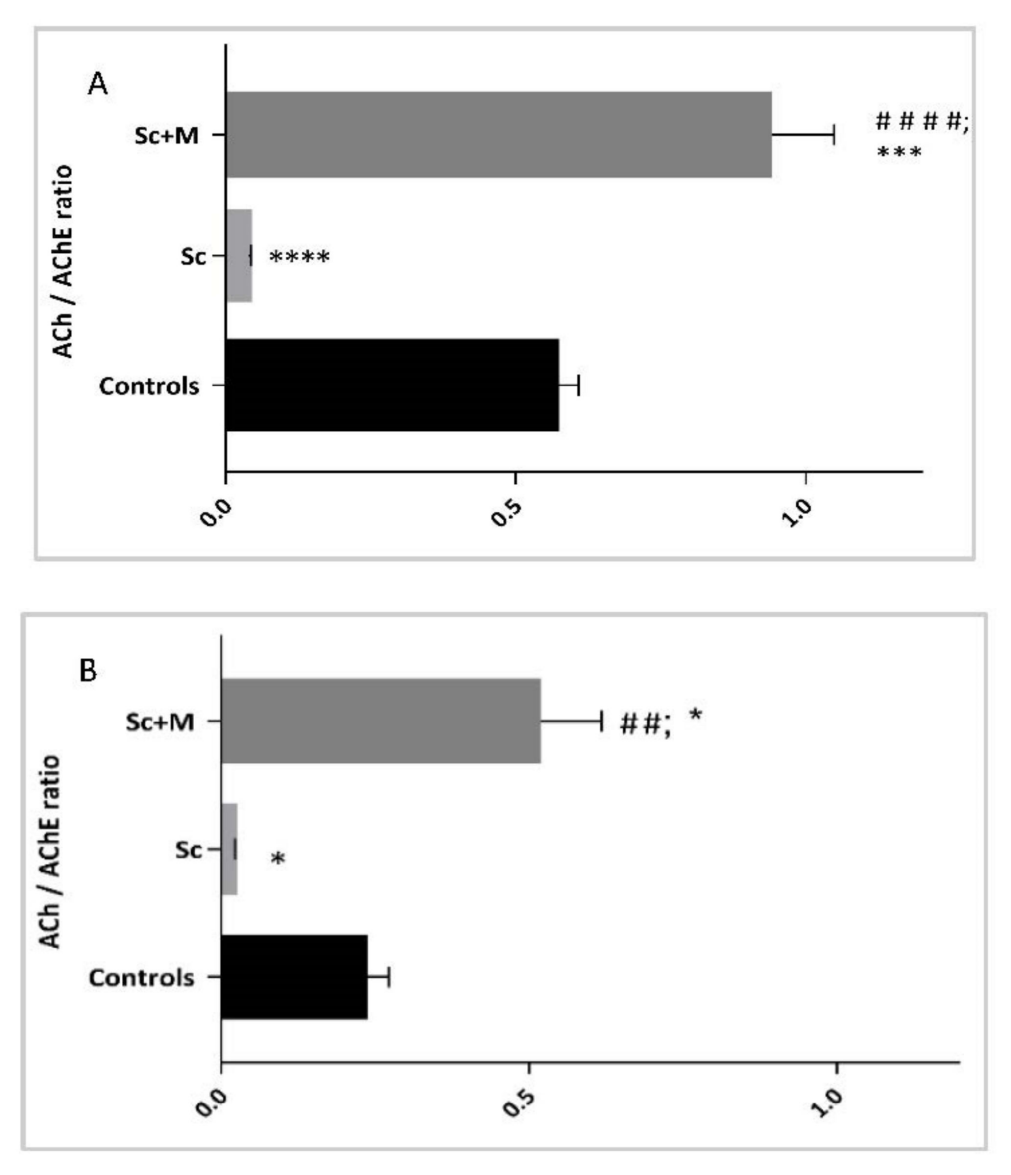
Cholinergic system influence index, calculated by the ratio of ACh levels to AChE activity in two brain regions—cortex (**A**) and hippocampus (**B**) connected to memory, in rats with scopolamine induced dementia, treated with Myrtenal (40 mg/kg) for 9 days; * *p* < 0.05, *** *p* < 0.001, **** *p* < 0.0001 vs. Controls; ^##^ *p* < 0.01, ^####^ *p* < 0.0001 vs. Sc group.

In the Sc-treated rats, the cholinergic influence index was significantly lower than the controls, with a 14-fold reduction in the cortex (*p* < 0.001) and an 11-fold decrease in the hippocampus (*p* < 0.01) (Figure 8).

**Figure 9:**
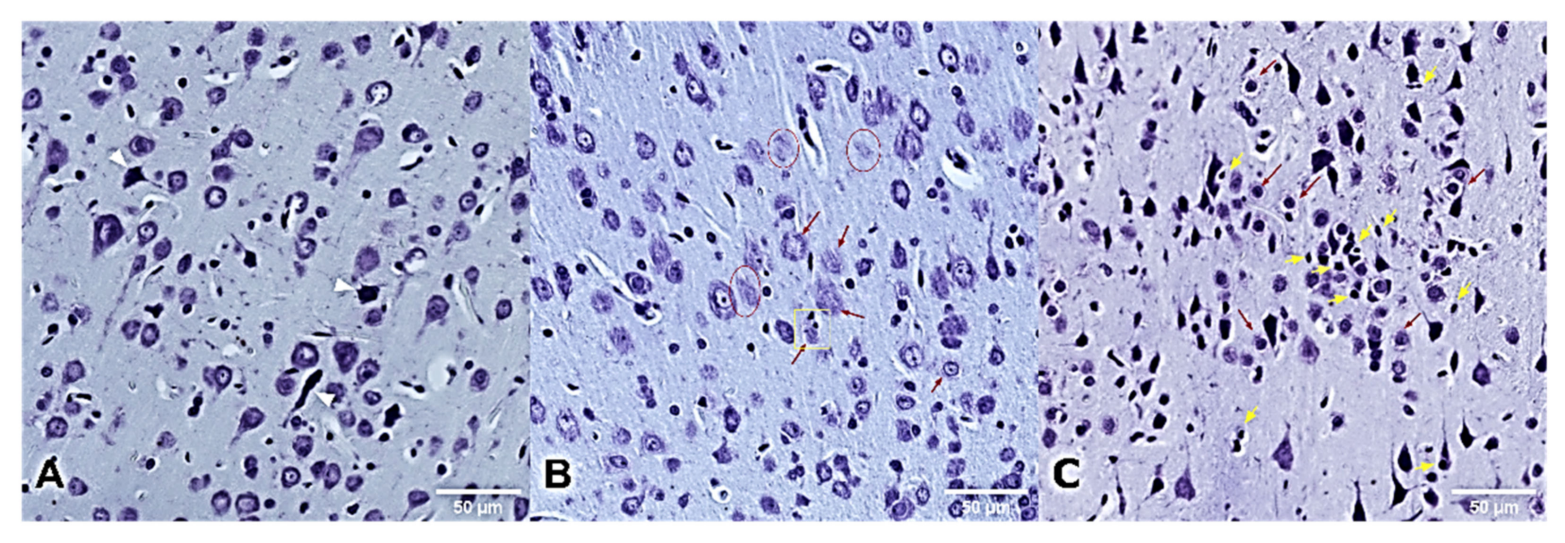
Histological abnormalities observed in the cerebral cortex of rats treated with scopolamine in the dose combination; Scale-bar—50 µm; (**A**) controls. Normal morphology of neurons in layer V of motor cortex (white arrows—artificial changes occurring post mortem, often mistaken for pathological); (**B**) scopolamine group. Chromatolysis (red arrows), neuron "shadows” (red ellipses) and gliosis in layer V of the retrosplenic cortex; (**C**) scopolamine group. Vacuolization and chromatolysis (red arrows) and solid gliosis (yellow arrows—glial cells) in layer III–IV of the sensory cortex (S1).

Myrtenal application significantly increased the cholinergic index. In the cortex of the myrtenal-treated animals, the ACh levels/AChE ratio reached a 23-fold increase (*p* < 0.001) vs. Sc group and a 67.9% increase (*p* < 0.01) as compared to the controls (Figure 9). In the hippocampus, the ratio ACh levels/AChE increased by 4-fold in the myrtenal group vs. the Sc group (*p* < 0.01) and by 119.1% (*p* < 0.05) as compared to the control group (Figure 8).

### 3.4. Histopathological Examinations

Morphological analysis of the cortex from Sc-treated rats revealed the presence of gliosis, neurons with fine vacuolation, and a marked loss of Nissl granulations (chromatolysis) (Figure 9). Neural shadows, areas of aggregation of microglia, as well as neurons with pyknosis or perineuronal microgliosis, signs of enhanced neuronal degeneration, were also present. These changes were most pronounced in the third and fifth layers of the neocortex (Figure 9).

**Figure 10:**
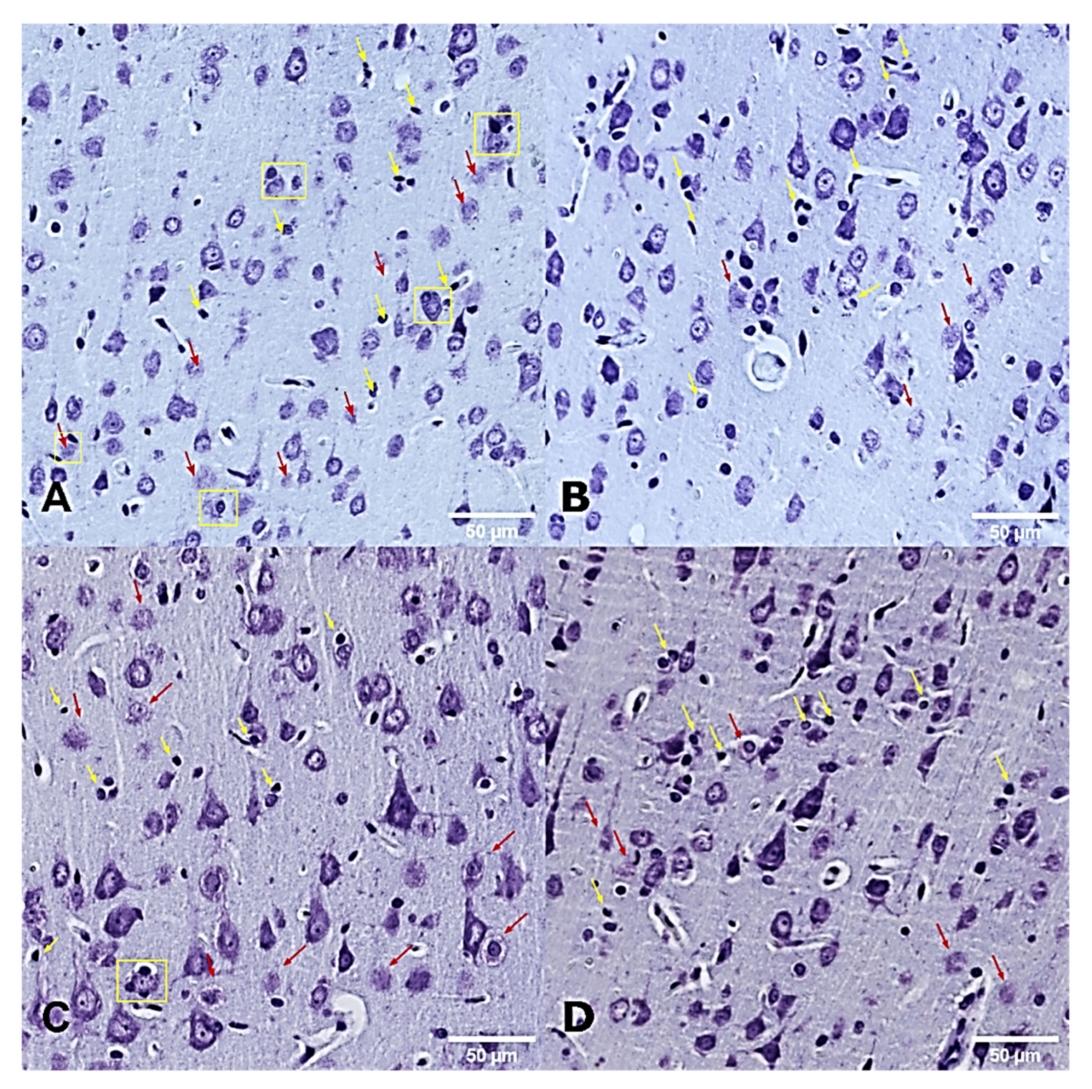
Examples of observed pathological changes in rats with scopolamine-induced dementia (dose modification)—comparison to the dementia group with that treated concomitantly with Myrtenal; (**A**) motor cortex layer V (M1) of a rat brain from the Sc group; (**B**) motor cortex layer V (M1) of a rat brain from Sc + M group; (**C**) sensory cortex layer V of a rat brain from the Sc group; (**D**)—Sensory cortex layer V of a rat brain from Sc + M group. (Scale bar—50 µm; Red arrows—neurons with signs of chromatolysis and/or vacuolization; yellow arrows—glial cells; yellow rectangle—perineuronal gliosis).

In the myrtenal-treated group, the signs of neuronal damage were reduced (Figure 10B,D). Karyolysis, karyopyknosis, and perineuronal microglycosis in brain tissue were relatively rare, as compared to the Sc group. However, cytoplasmic vacuolization and chromatolysis were observed.

**Figure 11:**
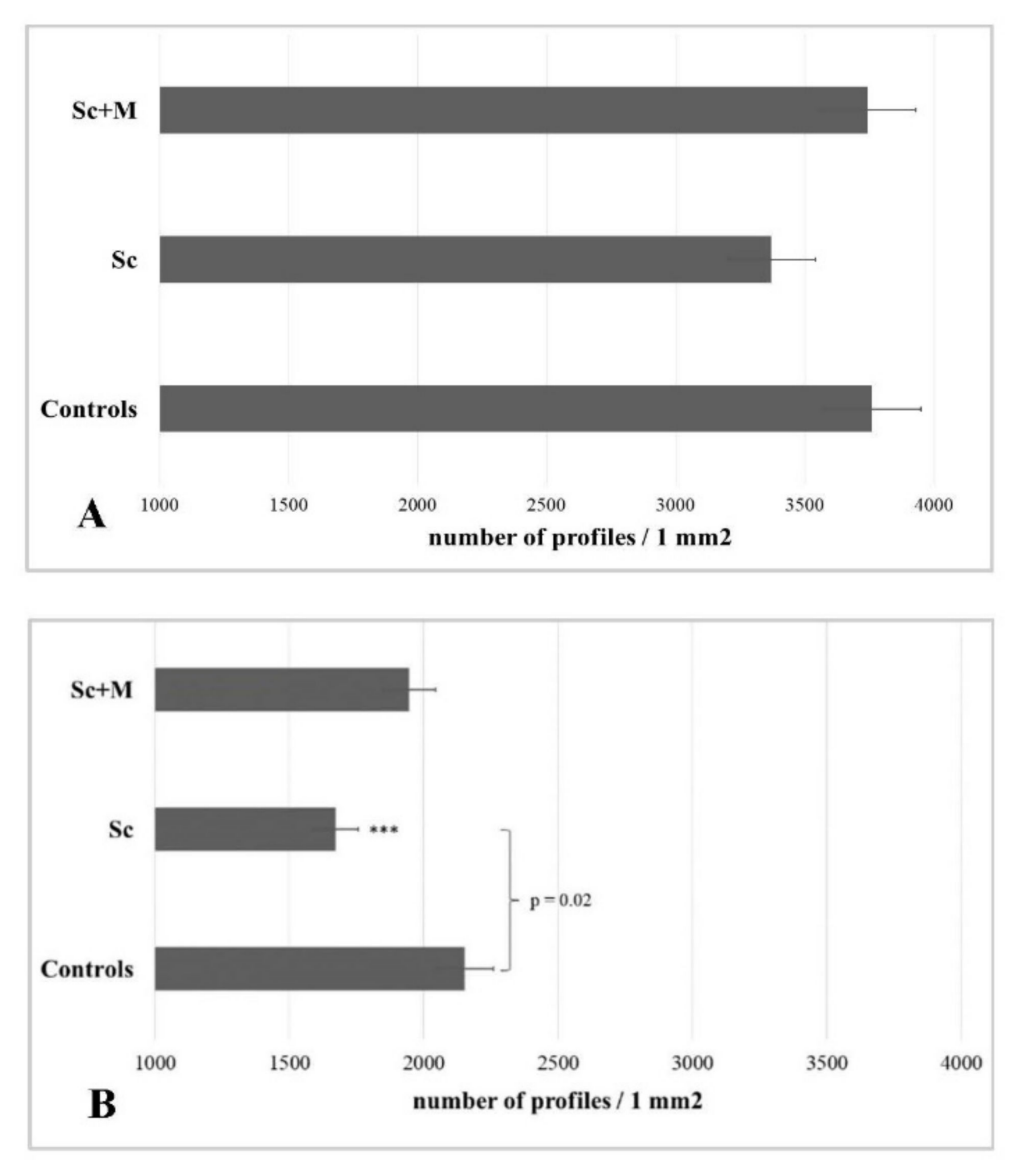
Effects on the total number of profiles per mm^2^ (density) (**A**) and on the number per mm^2^ of profiles (density) with caliper diameter greater than 6.5 µm (**B**) in brain cortex of rats with Scopolamine-induced dementia (dose modification) treated with myrtenal (40 mg/kg) for 9 days; *** *p* < 0.001 vs. Controls.

The quantitative analysis of the brain cortical density is presented in Figure 11.

The mean total profile density in controls was 3760 mm^−2^ (95% CI: 3429–4123 mm^−2^) and showed no significant differences from the Sc groups (Figure 11A). The density (number of profiles per 1 mm^2^) of all stained profiles absorbing more than 20% of light, showed no differences between the treatment groups. Myrtenal administration resulted in a 10.9% increase vs. the Sc group (*p* = n.s.).

The type of treatment had a significant effect on the mean density (number/mm^2^) of profiles with a diameter larger than 6.5 µm $(\chi^{2}=19.971;df=2\;;p=4.606*10^{-5}$). In this population of profiles, the damaging effect of Sc was manifested by a significant decrease in this measure by an average of about 500 profiles per mm^2^ compared to controls (*p* < 0.001, post hoc pairwise test with Holm adjustment) (Figure 11B). Rats treated with myrtenal were in an intermediate position. The profile density was decreased by approximately 200 profiles per mm^2^ as compared to the controls (*p* = 0.062; ns; post hoc pairwise test with Holm adjustment), but there was a significant increase of about 270 profiles per mm^2^ compared to the Sc group (*p* = 0.02). Although the density of “large” profiles in myrtenal-treated demented animals was reduced by 9.6% in comparison to the controls, a significant number of neurons (16.41%) compared to the Sc group was protected.

In the hippocampal formation, no significant changes in morphological and quantitative parameters were detected as a result of treatment with both scopolamine and its combination with M.

### 3.5. Summary Cluster Analysis of the Effects of Myrtenal on Scopolamine Induced Damage

**Figure 12:**
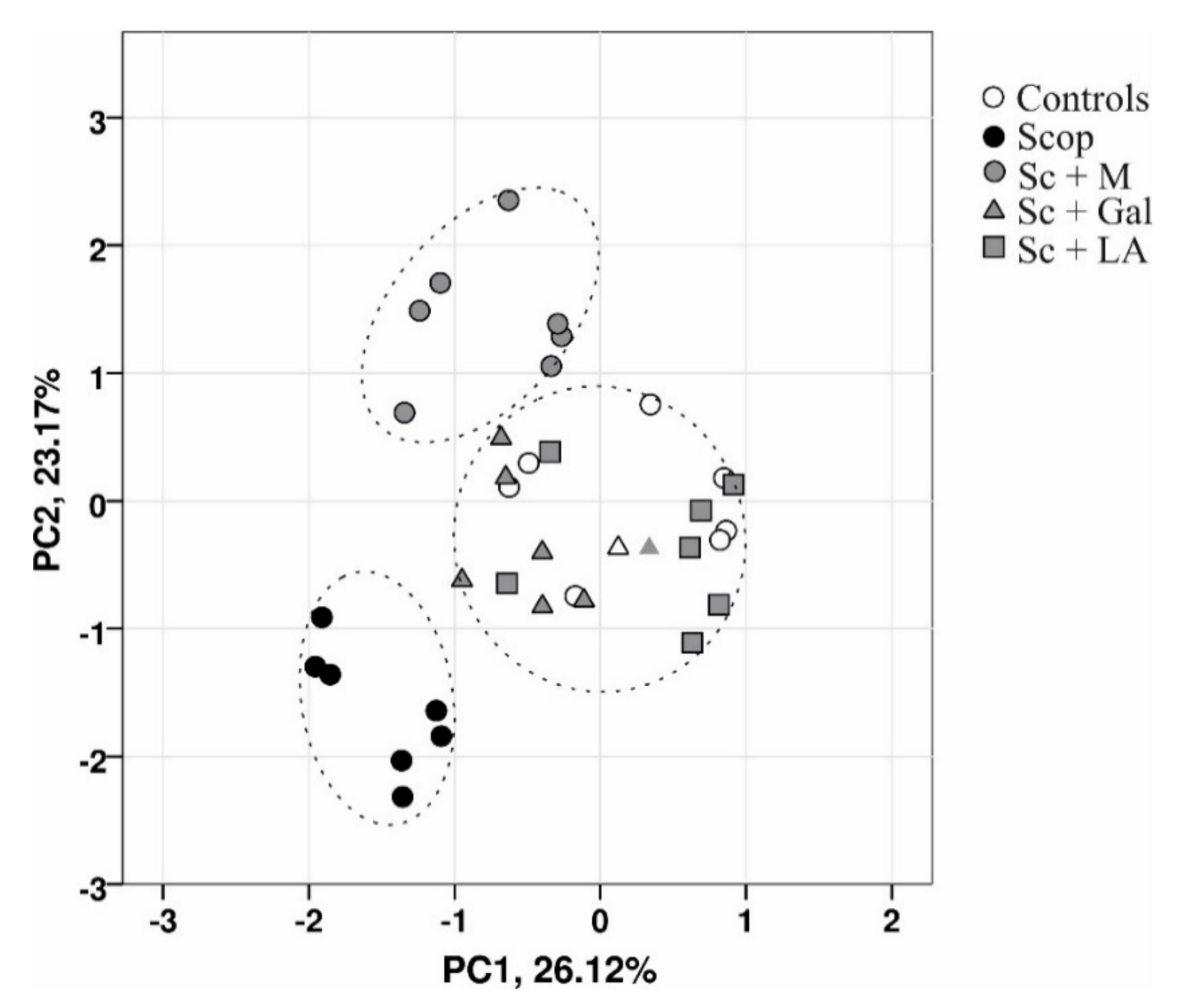
Scatter plot representation of the individual rat data points, using the first two principal components (PC1 and PC2) on a two-dimensional space, shows that the rats tend to cluster according to their treatment.

The overall effect of myrtenal, according to histological, biochemical, and behavioral data, on Sc-induced dementia, is presented in Figure 12.

The two reference treatments (Gal and LA) were clearly distinguished from the Sc group and gravitated toward the control group. On the other hand, myrtenal-treated demented rats formed an independent cluster. It was differentiated from controls but was also tangential to the rats treated with the reference compounds, suggesting to exert specific neuroprotective mechanism.

## 4. Discussion

Alzheimer’s disease is a complex neurodegenerative disorder, associated with cholinergic dysfunction, oxidative stress, memory, and behavioral impairment [42]. Despite the effort of the scientific community, until now there is no cure, and treatment is only symptomatic.

The search for new and more effective drugs continues. The combination of antioxidant properties, alongside the ability to affect the functions of the CNS, e.g., exerting AChE inhibitory properties, makes natural compounds very attractive potential candidates [43,44,45,46].

In this study, for the first time, we have investigated the neuroprotective effects of Myrtenal in an experimental model of dementia. Experimental dementia was produced by the treatment of rats with Sc, a widely used pharmacological agent [47].

**Scheme 1:**
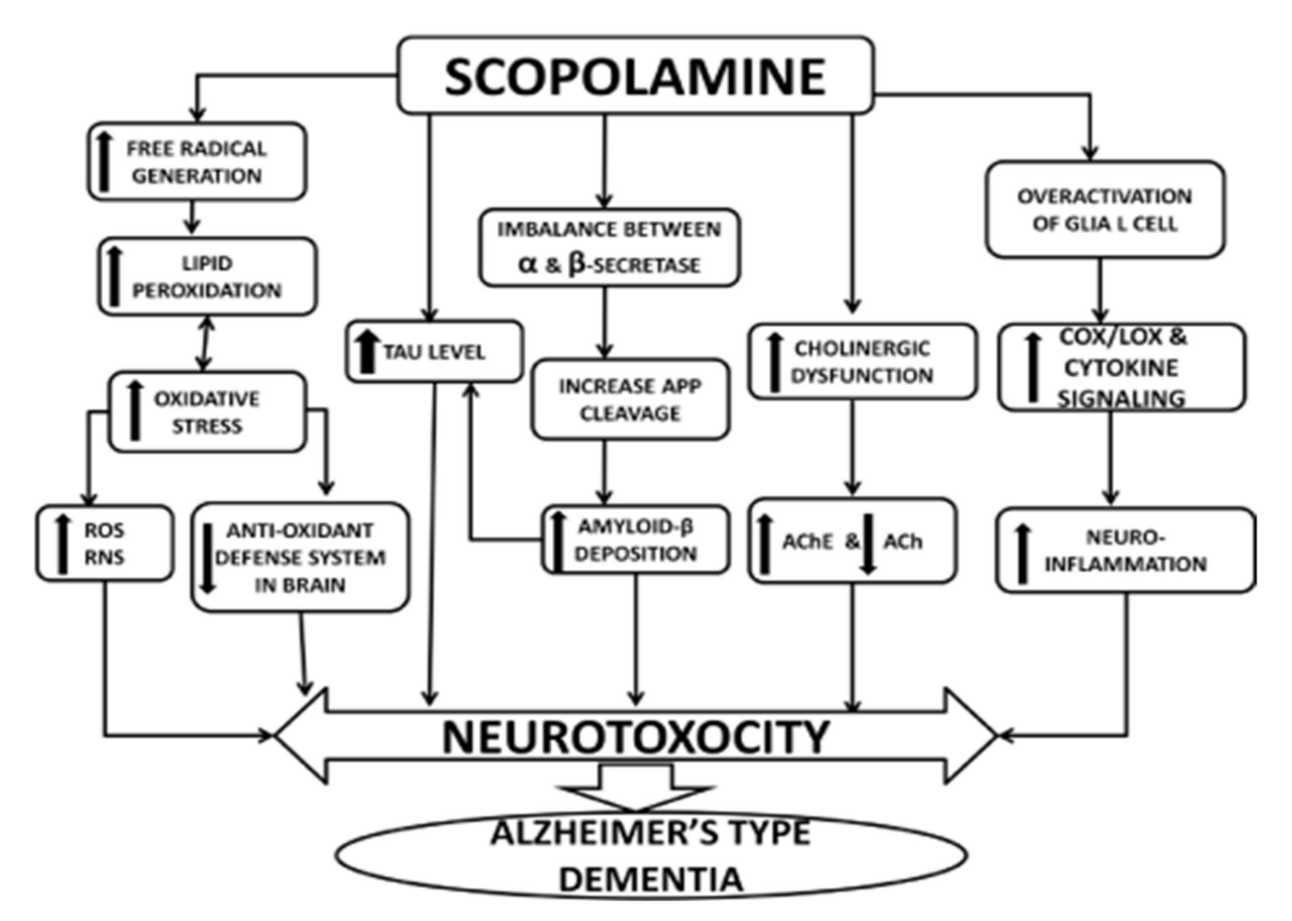
Damaging mechanisms of Scopolamine as a pharmacological agent used for inducing experimental dementia of Alzheimer’s type (Kaur et al., 2015). ↑-increase, ↓-decrease.

Even though Scopolamine has been used experimentally to induce memory impairment for many years, its mechanisms of action are not fully clarified. We believe that the following scheme (Scheme 1) published by Kaur et al. (2015) summarizes the known damaging mechanisms of Scopolamine, including tau levels and amyloid deposits [48]:

As a muscarinic cholinergic receptor antagonist, Sc impairs brain cholinergic transmission (increasing AChE activity, reducing ACh levels, and inhibiting choline acetyltransferase activity). It increases brain oxidative stress levels and affects the memory processes, inducing cognitive deficits similar to those observed in AD patients [49,50,51,52].

In the current work, the effect of the monoterpene myrtenal on recognition memory and habituation was evaluated. The novel object recognition test was used to assess changes in recognition memory of the experimental animals. Our results showed that multiple administrations of low-dose Sc caused disorientation and suppressed recognition memory of the experimental animals, manifested as a lack of clearly pronounced preference up to new objects. The recognition memory recovery effect was established in the LA-treated group. LA was used as a reference compound with antioxidant properties. This result demonstrates the key role of oxidative stress in memory impairment and the possibility of using antioxidant compounds for the treatment of mild cognitive dysfunction. Myrtenal-treated animals also showed a tendency for improving the Sc-induced damage in recognition memory.

The beneficial memory effect of myrtenal on rats with experimental dementia was confirmed in the open field test. According to the studies of Platel and Porsolt (1982) and Platel et al., (1984), the habituation of experimental animals placed in an unfamiliar environment is a behavioral expression of the activity of learning and memory processes [53,54]. Our results showed that after multiple Sc administration, the exploratory activity and habituation pattern of the animals were impaired, as shown by the increased delta cross index in comparison to the control. Myrtenal treatment decreased the delta cross index more than six times vs Sc (*p* < 0.001), indicating its ability in preventing memory impairment. Interestingly, the delta cross index in the above-mentioned group was lower than in the control, which can be explained by the already established anxiolytic effects of myrtenal [17].

According to many clinical and preclinical studies, the main factor for neurological disorders and related neurodegenerative diseases, including AD, is increased oxidative stress, confirmed by the findings that protein side chains are modified or directly damaged by reactive oxygen species (ROS), or from reactive nitrogen species (RNS), or indirectly from lipid peroxidation products [55]. Progression of neurodegenerative diseases is accompanied by changes in activity and expression of antioxidant enzymes [56].

The ability of Sc to induce oxidative stress in the brain as part of its memory impairment effect is well known [57,58,59,60,61,62]. This includes increased LPO production, decreased tGSH concentration, and changed SOD activity in the brain of rats with experimental dementia [63].

Myrtenal application partly restored the main oxidative stress markers induced by the Sc and the effect was commensurable with those of the reference compound, LA.

In order to determine if the beneficial memory effect of myrtenal was mediated by the modulation of the cholinergic system, its effect on ACh brain levels and AChE activity in the cortex and hippocampus of dementia rats were evaluated.

In our experimental model of dementia, multiple Sc administrations affected the cholinergic system by increasing AChE activity followed by a reduction in ACh levels and the inhibition of choline acetyltransferase (ChAT) activity [64,65,66]. Our results showed that myrtenal administration significantly increased ACh brain levels in Sc-treated animals, entailing a stronger effect than the reference drug, Gal, although AChE activity was not significantly affected. Docking analysis confirmed this observation, showing that myrtenal could bind to the AChE catalytic center to a lesser extent than Gal. This result gave us a reason to believe that myrtenal could realize its effect on the cholinergic system by affecting ChAT enzyme activity. This hypothesis is supported by the observation that α-pinene, which metabolite is myrtenal, possesses ChAT excitatory effect [12], Our cholinergic system index calculations also confirmed this. They were similar to those reported by Karami et al., (2019) [67], where the ratio of ChAT to AChE in CSF was defined as the cholinergic system influence index. It was proposed to measure this index in AD patients in order to assess the treatment effect. An increase in this ratio means an increase in ACh levels. In our case, an increase in the ratio ACh levels/AChE activity means an increase in ACh levels, resulting from stimulation of cholinergic transmission. Provided that AChE activity is not altered by myrtenal, we consider this to be an indirect indication that this process is occurring by the participation of the enzyme ChAT.

Alongside the identified neuromodulator properties and the observed positive effects of the test substance on the recognition memory and habituation of experimental animals, histopathological studies also indicated that myrtenal has the ability to antagonize the damaging action of Sc.

In the morphological analysis of brain cortex in rats treated with Myrtenal, the magnitude of glial reaction appeared similar to the Sc, but the observed signs of cortical degeneration were more moderate. The main difference with respect to the demented rats was that the karyolysis, karyopyknosis, and perineuronal microgliosis in the brain cortical tissue were relatively rare. These histological findings were confirmed with quantitative analysis—the density of cell profiles in animals treated with myrtenal was reduced compared to healthy controls, but there was a significant increase as compared to the Sc group. Sc significantly reduced the mean density of provisional vital neurons in the cerebral cortex.

In rodents treated with myrtenal concomitantly with Sc, the impairment was less pronounced and the number of viable cells in this group was markedly increased compared to the demented group of rats (*p* < 0.05). Cortical changes showed a decreasing trend in the volume of the Nissl substance and/or the number of neurons per unit area in animals with induced dementia as rats treated with myrtenal tended to reduce these negative effects. No significant differences similar to those found in the cortex were detected in the hippocampus.

## 5. Conclusions

For the first time, we have shown a positive effect of myrtenal on impaired memory in rats with an experimental model of dementia. The histological data established that the neuroprotective effect of myrtenal mainly pertained to the cerebral cortex. In addition, our data suggest that the beneficial effects of myrtenal are related to its antioxidant action, combined with the stimulating effect on brain cholinergic neurotransmission.

## Data Availability

Data is contained within the article.
